# Quinoa Response to Application of Phosphogypsum and Plant Growth-Promoting Rhizobacteria under Water Stress Associated with Salt-Affected Soil

**DOI:** 10.3390/plants11070872

**Published:** 2022-03-24

**Authors:** Moshira A. El-Shamy, Tarek Alshaal, Hossam Hussein Mohamed, Asmaa M. S. Rady, Emad M. Hafez, Abdullah S. Alsohim, Diaa Abd El-Moneim

**Affiliations:** 1 Crop Intensification Research Department, Field Crops Research Institute, Giza 12511, Egypt; moshiraahmed8@gmail.com; 2 Department of Applied Plant Biology, University of Debrecen, Böszörményi Street 138, 4032 Debrecen, Hungary; tarek.ibrahim@agr.kfs.edu.eg; 3 Soil and Water Department, Faculty of Agriculture, University of Kafrelsheikh, Kafr El-Sheikh 33516, Egypt; 4 Department of Agronomy, Faculty of Agriculture, Ain Shams University, Cairo 11782, Egypt; dr.hossam16@yahoo.com; 5 Crop Science Department, Faculty of Agriculture (EL-Shatby), Alexandria University, Alexandria 21545, Egypt; asmaa.mohamed@alexu.edu.eg; 6 Department of Agronomy, Faculty of Agriculture, Kafrelsheikh University, Kafr El-Sheikh 33516, Egypt; 7 Department of Plant Production and Protection, College of Agriculture and Veterinary Medicine, Qassim University, Burydah 51452, Saudi Arabia; 8 Department of Plant Production (Genetic Branch), Faculty of Environmental Agricultural Sciences, Arish University, Arish 45511, Egypt; dabdelmoniem@aru.edu.eg

**Keywords:** soil amendments, drought, soil salinity, quinoa, antioxidant enzymatic activity

## Abstract

The aim of the study was to estimate the impact of soil amendments (i.e., phosphogypsum and plant growth-promoting rhizobacteria (PGPR)) separately or their combination on exchangeable sodium percentage (ESP), soil enzymes’ activity (urease and dehydrogenase), pigment content, relative water content (RWC), antioxidant enzymatic activity, oxidative stress, productivity, and quality of quinoa under deficient irrigation conditions in two field experiments during the 2019–2020 and 2020–2021 seasons under salt-affected soil. Results revealed that ESP, soil urease activity, soil dehydrogenase activity, leaf chlorophyll a, b, and carotenoids, leaf K content, RWC, SOD (superoxide dismutase), CAT (catalase), and POD (peroxidase) activities were declined, resulting in overproduction of leaf Na content, proline content, and oxidative stress indicators (H_2_O_2_, malondialdehyde (MDA) and electrolyte leakage) under water stress and soil salinity, which negatively influence yield-related traits, productivity, and seed quality of quinoa. However, amendment of salt-affected soil with combined phosphogypsum and seed inoculation with PGPR under deficient irrigation conditions was more effective than singular application and control plots in ameliorating the harmful effects of water stress and soil salinity. Additionally, combined application limited Na uptake in leaves and increased K uptake and leaf chlorophyll a, b, and carotenoids as well as improved SOD, CAT, and POD activities to ameliorate oxidative stress indicators (H_2_O_2_, MDA, and electrolyte leakage), which eventually positively reflected on productivity and quality in quinoa. We conclude that the potential utilization of phosphogypsum and PGPR are very promising as sustainable eco-friendly strategies to improve quinoa tolerance to water stress under soil salinity.

## 1. Introduction

Quinoa (*Chenopodium quinoa* Willd.) is a seed crop of the Chenopodiaceae family belonging to the C3 group of plants [1]; it has the capability to adapt to diverse agro-ecosystems beginning from sea level to nearly 4000 m in elevation [2]. Quinoa is deemed as a multipurpose agricultural crop with great genetic diversity [3]. Scientific research started to focus on conducting experiments on quinoa recently. The United Nations in 2013 announced the International Year of Quinoa to offer food security [4] and quinoa has received globally trustworthy attention due to its tolerance to harsh environmental conditions, resulting in increasing the number of countries cultivating quinoa since then. The seeds can be consumed for human food and the crop residues may be utilized for animal feed, in addition to several valuable compounds such as saponin, oil, and protein concentrate [5]. The high nutritional value of the quinoa plant is largely owing to its grain, which is exceptionally rich in proteins and essential amino acids, mainly lysine (5.1–6.4%) and methionine, dietary fiber and minerals (Ca, P, K, Fe, Zn and Mg), as well as being rich in vitamins and poly-unsaturated fatty acids [6]. Quinoa is known as a pseudo-grain and its grains are an achene type similar to cereals that can be ground into flour and used as a grain crop, although it does not belong to the Poaceae botanical family [7]. Recent scientific literature has confirmed quinoa’s potentiality to be an alternative to cereals such as wheat, rye, and barley [8]; however, it is necessary to emphasize that quinoa is still a niche market being consumed by more informed and conscious consumers. Nevertheless, the production of quinoa crop is very insufficient to face the ever-increasing demand under abiotic stresses, especially water stress and salt-affected soil in arid and semi-arid regions.

In the Mediterranean zone and arid and semi-arid regions, salt-affected soil has significantly damaged the agricultural soils worldwide resulting in low soil quality, crop development, and productivity [9]. Roughly seven percent of the world land area and less than 25 percent of agricultural lands are influenced by high soil salinity in addition to low quality water resources, which cause soil salinization [10]. Soil quality is deteriorated owing to high amounts of salt in the soil, which leads to clay dispersion, low hydraulic conductivity, and low water leakage into the soil as well as increases in Na content in the soil solution [11]. Consequently, salt-affected soil impedes morphological and physiological attributes such as reduced plant development [12], enzymatic activity [13], chlorophyll content, relative water content, and grain yield whilst it increases oxidative stress and ion imbalance [14]. Therefore, some alternative agricultural mechanisms are required to combat the damaging effect of salt stress, which have eco-friendly and economic benefits for farmers.

Field crops often face numerous abiotic stresses, which harmfully influence its growth and productivity, such as water stress that has deleterious impacts on the plant development [15]. Environmental stressors, such as water stress, result in various morphological and physiological disturbances in plants including ion imbalance, pigments, relative water content by overproduction of oxidative stress that leads to reactive oxygen species during respiration, and photosynthetic processes in plants resulting in cell death [16]. Antioxidant enzymatic activities including superoxide dismutase (SOD), ascorbate (APX), peroxidase (POD), and catalase (CAT) have a potential impact as a defense mechanism in ameliorating the harmful impacts of drought-induced oxidative stress and scavenge ROS [17]. Water resources gradually reduce owing to the augmented request for water by increasing the population and water consumption for different purposes. Consequently, the field crops will indeed face a severe water stress [18]. Therefore, there is an urgent need to discover alternative technologies that are eco-friendly and efficient to increase plant tolerance against water stress [19,20].

Phosphogypsum is a new alternative approach to inorganic chemicals. Phosphogypsum (PG) is a by-product of the phosphate fertilizer industry, due to the manufacturing of phosphoric acid from rock phosphate (fluorapatite). Globally, around 160 Mt of phosphogypsum are manufactured yearly and it is mainly removed in big stocks or discharged into waterways [21]. It comprises principally sulfur, calcium oxide, and minor quantities of phosphorous [22] and aluminum (Al), iron (Fe), silicon (Si), and magnesium (Mg^2+^) [23]. Because the dangerous impacts of phosphogypsum waste in the ecosystem are expanding owing to unlimited industrialization, appropriate administration is needed to diminish the opposing impacts on humans and the environment [24]. Worldwide, phosphogypsum is applied in agriculture either as a soil amendment (CaSO_4_) or as fertilizer [25], which has a potentiality in improving alkaline soils by the improvement of soil physical and chemical characterization and the availability of various nutrients to crops (direct) owing to its high content of calcium (Ca^2+^), P, and sulfur (S). It helps in augmenting aeration, water holding capacity, oxygen penetration, and nutrient uptake in soil or when coupled with microbes to improve the solubility of nutrients (indirect) [26] owing to its ability to maintain a low pH level, as well as exclude Na^+^ and augment the concentration of osmolytes such as K^+^ within leaves [26]. Various authors have proposed that the coupled application of phosphogypsum and microbial inoculation augmented the levels of NPK absorption and increased the growth of maize plants [27], thereby increasing the microbial biomass carbon, N contents, soil enzyme, and plant growth [27].

Inoculation of crop plants with beneficial microbes (plant growth-promoting rhizobacteria and PGPR) has the potential to enhance soil–water–plant relations [28,29] and ameliorate soil salinity [30] through the ability of soil microbes to manipulate phytohormonal signaling and release an enzyme 1-aminocyclopropane-1-carboxylic acid (ACC)-deaminase, produce exopolysaccharide (EPS), and increase the plant antioxidant enzymes that can have the ability to ameliorate the harmful impact of abiotic stress on root development [31]. PGPR inoculation has the potential to achieve nitrogen fixation, phosphate solubilization, and the release of nutrients from organic and inorganic forms to a bioavailability form that seems to be a promising treatment for improving the tolerance of crops to water stress and soil salinity [32]. Several researchers have suggested that PGPR application is the most useful and eco-friendly approach to increment the crop productivity along with improving the soil health in the long term in stressed soil [33].

The aim of the study was to include phosphogypsum by-product combined with plant growth-promoting rhizobacteria under water stress in salt-affected soil and enhance soil characteristics; this may, in turn, be beneficial as an environmentally friendly approach to increase quinoa productivity and ameliorate the soil properties.

## 2. Results

### 2.1. Exchangeable Na % in the Soil and Soil Enzymes’ Activity

Table 1 displays that irrigating quinoa plants by deficit irrigation under saline soil increased ESP compared to regular irrigation. However, the application of phosphogypsum, PGPR, or their interaction significantly declined it regardless of the irrigation treatments in both seasons, i.e., 2019–2020 and 2020–2021 (Table 1). The lowest ESP was obtained for experimental soil that received phosphogypsum and PGPR under regular irrigation. Remarkably, applying phosphogypsum and PGPR to soil irrigated with deficit irrigation treatment displayed lower ESP than control soil that received regular irrigation. PGPR displayed a higher positive impact on ESP than phosphogypsum.

The activity of soil urease (mg TPF g^−1^ soil d^−1^) and dehydrogenase (mg NH_4_^+^-N g^−1^ soil d^−1^) enzymes was significantly (*p* < 0.05) reduced upon irrigating quinoa plants with deficit irrigation in saline soil during 2019–2020 and 2020–2021 (Table 1). However, the addition of phosphogypsum, PGPR, or both significantly mitigated the harmful impact of deficit irrigation in saline soil on enzymes’ activity. For example, the addition of phosphogypsum and PGPR significantly increased the activity of dehydrogenase after deficit irrigation compared to regular irrigation (control) in absence of phosphogypsum or PGPR application, or both. The highest urease activity was measured in the soil irrigated with regular irrigation that received phosphogypsum and PGPR. Sole application of PGPR stated a higher positive effect on urease enzyme than the sole application of phosphogypsum (Table 1). Similarly, dehydrogenase activity under deficit irrigation was the lowest for untreated plants (control) during 2019–2020 and 2020–2021. Dehydrogenase activity was increased by the sole application of phosphogypsum or PGPR; however, the application of phosphogypsum and PGPR further increased the activity of dehydrogenase during 2019–2020 and 2020–2021 (Table 1). Moreover, phosphogypsum and PGPR resulted in an augment in dehydrogenase activity when plants were irrigated with deficit irrigation compared to control plants irrigated with regular irrigation or treated with phosphogypsum only in saline soil during 2019–2020 and 2020–2021.

### 2.2. Concentration of Na^+^, K^+^, and K^+^/Na^+^ in the Leaves

Dropping the irrigation level in quinoa plants from regular irrigation to deficit irrigation caused an increment in Na^+^ content and a decrease in K^+^ content in quinoa leaves relative to the plants grown under the untreated plots (control) (Table 2). Deficit irrigation resulted in an increment in Na^+^ content in leaves whereas, for K^+^ content, the same deficit irrigation declined the K^+^ content compared to the control of regular irrigation. Opposingly, the soil applications with phosphogypsum and PGPR altered the ionic equilibrium in favor of potassium, where Na^+^ content decreased for phosphogypsum and PGPR under regular irrigation, also K^+^ content increased for phosphogypsum and PGPR under regular irrigation. The lowest Na^+^ content was found under the combined application of phosphogypsum and PGPR; the coupled treatment was more effective, which declined the Na^+^ content lower than the control of deficit irrigation. Phosphogypsum had a higher impact than PGPR on augmenting K^+^ content. However, the most significant boost of K^+^ was in quinoa grown with the coupled application of phosphogypsum and PGPR, irrespective of irrigation treatment, where under deficit irrigation treatment, the coupled application resulted in an increment over its control and over the control of regular irrigation (Table 2).

### 2.3. Photosynthetic Pigments (Chlorophyll a, Chlorophyll b, and Total Carotenoids)

Under saline soil, deficit irrigation treatment declined the levels of photosynthetic pigments, and soil application using phosphogypsum and PGPR improved significantly (*p* < 0.05) the levels of chlorophyll a, chlorophyll b, and total carotenoids (Table 3) during 2019–2020 and 2020–2021 seasons. In control plants, deficit irrigation significantly (*p* < 0.05) declined the levels of chlorophyll a, chlorophyll b, and total carotenoids during 2019–2020 and 2020–2021 seasons. Quinoa plants had the highest levels of photosynthetic pigments when irrigated well. Under regular irrigation, the combined application of phosphogypsum and PGPR together had the highest levels of chlorophyll a, chlorophyll b, and total carotenoids during 2019–2020 and 2020–2021 seasons, followed by phosphogypsum alone, then PGPR. Although all photosynthetic pigments were significantly (*p* < 0.05) reduced under deficit irrigation treatment, the coupled application of phosphogypsum and PGPR had the highest levels of chlorophyll a, chlorophyll b, and total carotenoids during 2019–2020 and 2020–2021 seasons.

### 2.4. Proline Content, Relative Water Content, and Electrolytic Leakage

**Figure 1:**
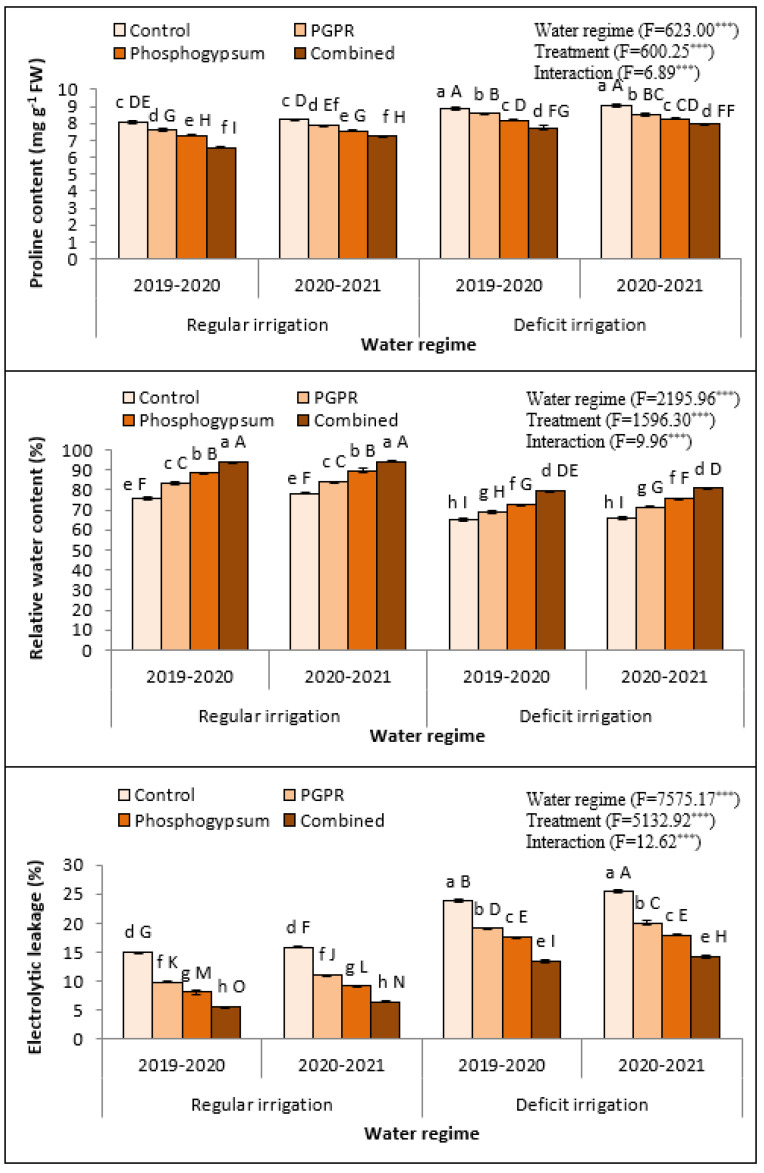
Response of proline content, relative water content, and electrolyte leakage in quinoa (*Chenopodium quinoa*) to different water regimes in the presence of singular or dual application of PGPR and phosphogypsum during two growing seasons (2019–2020 and 2020–2021). Different lowercase letters on the same columns of the same season are significant according to the Tukey’s test (*p* ≤ 0.05) and different uppercase letters are significant regardless of the growing season according to the Tukey’s test (*p* ≤ 0.05). Data are Means ± SD and n = 3. *** denotes significance at *p* ≤ 0.001.

Soil application of phosphogypsum and PGPR significantly reduced the proline content and electrolyte leakage, while it increased RWC of deficiently irrigated Quinoa plants under saline soil conditions. The impact of soil application by phosphogypsum and PGPR on the response of quinoa plants to abiotic stresses, including proline content, relative water content, and electrolyte leakage, were investigated during the 2019–2020 and 2020–2021 seasons (Figure 1). Although the irrigation deficiency considerably increased proline content in both seasons, soil application using phosphogypsum, PGPR, or both, significantly declined proline content in regularly irrigated quinoa plants as well as decreased it in quinoa leaves under deficient irrigation in 2019–2020 and 2020–2021. RWC of quinoa leaves declined under irrigation deficiency during the 2019–2020 and 2020–2021 seasons (Figure 1). However, soil application of phosphogypsum and PGPR enhanced the RWC of quinoa leaves during irrigation deficiency and when regularly irrigated. The combined application of phosphogypsum and PGPR together increased the RWC of quinoa leaves during the 2019–2020 and 2020–2021 seasons (Figure 1). In contradiction with RWC, electrolyte leakage (EL) of fully expanded flag leaves of osmotic-stressed quinoa plants increased under irrigation deficiency. Furthermore, dual phosphogypsum and PGPR application appreciably declined the electrolyte leakage of quinoa flag leaves at both irrigation treatments during the 2019–2020 and 2020–2021 seasons (Figure 1).

### 2.5. Phosphogypsum and PGPR Improved the Antioxidant-Related Enzymatic Activity in Deficiently Irrigated Quinoa Plant under Saline Soil

**Figure 2:**
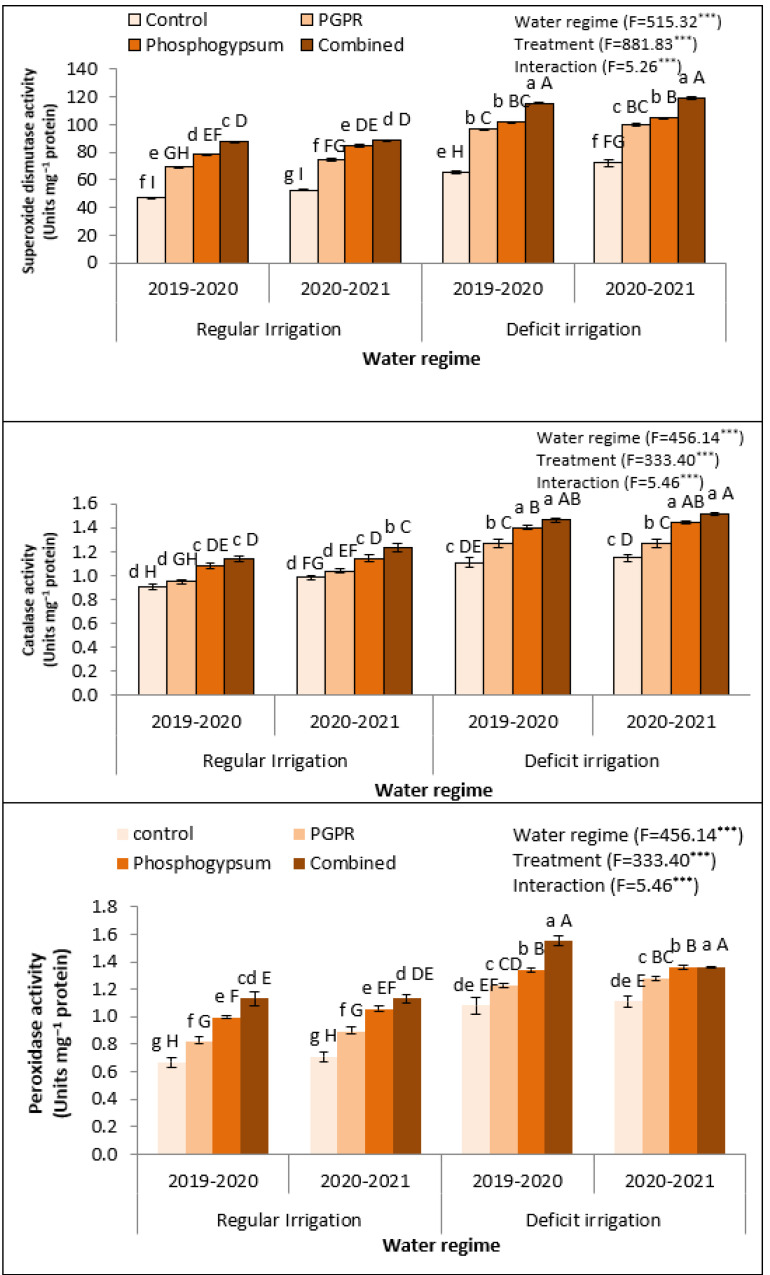
Response of some antioxidant enzymes in quinoa (*Chenopodium quinoa*) to different water regimes in the presence of singular or dual application of PGPR and phosphogypsum during two growing seasons (2019–2020 and 2020–2021). Different lowercase letters on the same columns of the same season are significant according to the Tukey’s test (*p* ≤ 0.05) and different uppercase letters are significant regardless of the growing season according to the Tukey’s test (*p* ≤ 0.05). Data are Means ± SD and n = 3. *** denotes significance at *p* ≤ 0.001.

To better understand how soil treatments by phosphogypsum and PGPR mitigate the oxidative stress in deficiently irrigated quinoa plants, the enzymatic activities of three antioxidant enzymes including superoxide dismutase (SOD), catalase (CAT), and peroxidase (POD) have been colorimetrically assessed during two growing seasons (2019–2020 and 2020–2021), as shown in Figure 2. Quinoa plants exposed to deficient irrigation in salt-affected soil significantly augmented antioxidant enzymes’ activity (Figure 2). Higher activity of SOD, CAT, and POD were attained in the quinoa leaves of control plants under deficient irrigation as compared to the regular irrigation. Deficient irrigation was more effective than the application of phosphogypsum or PGPR alone or in a combination form, where it increased the enzymatic activity. For example, under deficient irrigation, application of phosphogypsum and PGPR increased SOD activity over control of regularly irrigation (Figure 2). The same performance was recorded for CAT and POD, where phosphogypsum increased CAT activity over its control while the control of deficient irrigation recorded an increase over regular irrigation (Figure 2). The most support for the activity of antioxidant enzymes was noted for the coupled application (phosphogypsum and PGPR), which increased the activity of SOD compared with the control. Although the activities of antioxidant enzymes were increased under the application of phosphogypsum and PGPR under deficient irrigation or regular irrigation conditions, the increase in activity level was not higher enough under deficient irrigation. This was particularly the case for POD activity, where the application of phosphogypsum and PGPR recorded the maximum activity of POD under deficiently irrigated conditions (Figure 2).

### 2.6. Phosphogypsum and PGPR Application Deflate-Stress Biochemical Indicators in Stressed Quinoa Plants

**Figure 3:**
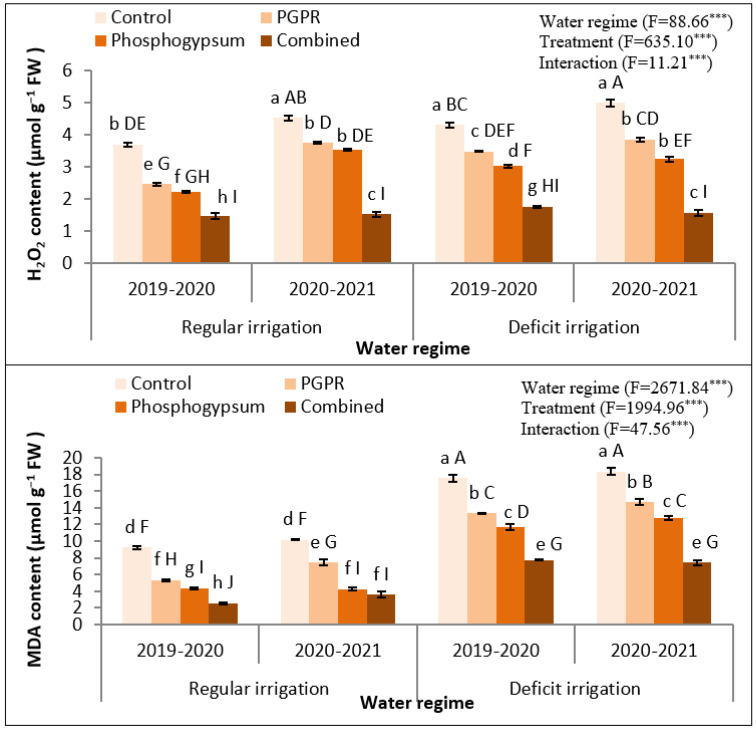
Response of hydrogen peroxide (H_2_O_2_) and lipid peroxide (MDA) in quinoa (Chenopodium quinoa) to different water regimes in the presence of singular or dual application of PGPR and phosphogypsum during two growing seasons (2019–2020 and 2020–2021). Different lowercase letters on the same columns of the same season are significant according to the Tukey’s test (*p* ≤ 0.05) and different uppercase letters are significant regardless of the growing season according to the Tukey’s test (*p* ≤ 0.05). Data are Means ± SD and n = 3. *** denotes significance at *p* ≤ 0.001.

Deficient irrigation noticeably increased the stress biochemical indicators as confirmed by H_2_O_2_ and MDA in 2019–2020 and 2020–2021 seasons (Figure 3). Coupled soil application of phosphogypsum and PGPR significantly (*p* < 0.05) reduced the accumulation of both molecules. Concisely, MDA levels significantly (*p* < 0.05) declined, owing to the soil application by phosphogypsum, PGPR, or both, during 2019–2020 and 2020–2021 seasons. Similarly, H_2_O_2_ levels were declined in 2019–2020 and 2020–2021 seasons (Figure 3); however, this decline was significant (*p* < 0.05) in both growing seasons.

### 2.7. Phosphogypsum and PGPR Application Enhanced the Yield Traits in Stressed Quinoa Plants

Deficient irrigation harmfully modified the yield of stressed quinoa including plant height (Table 4), 1000-seed weight (Table 4), seed yield (Table 4), foliage yield (Table 5), biological yield (Table 4), and harvest index (Table 4). However, soil application using phosphogypsum and PGPR significantly increased the quinoa yield. Concisely, the dual application of phosphogypsum and PGPR to regularly irrigated soils produced the highest plant height (192.7 ± 0.12 and 209.5 ± 0.27 cm), 1000-seed weight (4.91 ± 0.104 and 5.21 ± 0.027 gm), seed yield (3.58 ± 0.084 and 3.87 ± 0.015 t ha^−1^), foliage yield (5.08 ± 0.090 and 5.37 ± 0.015 t ha^−1^), biological yield (8.67 ± 0.050 and 7.96 ± 0.072 t ha^−1^), and harvest index (41.3 ± 0.33 and 40.6 ± 0.09%) in the 2019–2020 and 2020–2021 seasons, respectively. Similarly, the combined application of phosphogypsum and PGPR to deficiently irrigated soils produced the second highest plant height (120.5 ± 0.44 and 126.1 ± 0.38 cm;), 1000-seed weight (2.50 ± 0.012 and 2.35 ± 0.035 g), seed yield (2.65 ± 0.049 and 2.73 ± 0.085 t ha^−1^), foliage yield (4.15 ± 0.049 and 4.23 ± 0.085 t ha^−1^), biological yield (6.79 ± 0.099 and 6.95 ± 0.170 t ha^−1^), and harvest index (39.0 ± 0.16 and 39.2 ± 0.24%)) in the 2019–2020 and 2020–2021 seasons, respectively. Control quinoa plants had the lowest plant height (Table 5), 1000-seed weight (Table 4), seed yield (Table 4), foliage yield (Table 4), biological yield (Table 4), and harvest index (Table 4) during both seasons.

### 2.8. Phosphogypsum and PGPR Application Enhanced the Seed Quality in Stressed Quinoa Plants

**Figure 4:**
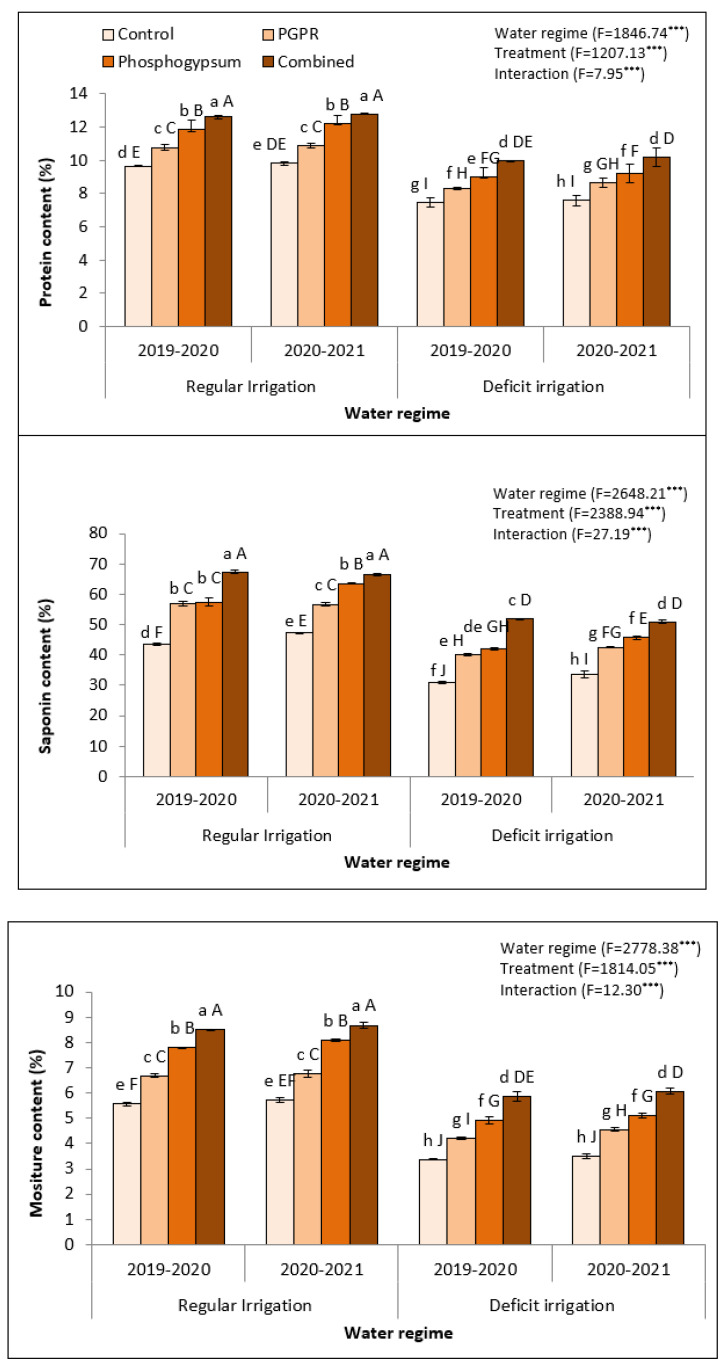
Variations in seed protein, saponin, and moisture contents in quinoa (*Chenopodium quinoa*) exposed to different water regimes in the presence of singular or dual application of PGPR and phosphogypsum during two growing seasons (2019–2020 and 2020–2021). Different lowercase letters on the same columns of the same season are significant according to the Tukey’s test (*p* ≤ 0.05) and different uppercase letters are significant regardless of the growing season according to the Tukey’s test (*p* ≤ 0.05). Data are Means ± SD and n = 3. *** denotes significance at *p* ≤ 0.001.

Deficient irrigation harmfully decreased the yield of stressed quinoa including protein content, moisture content, and saponin content in seeds of quinoa (Figure 4). However, soil application using phosphogypsum and PGPR significantly increased the quinoa yield. Concisely, the coupled application of phosphogypsum and PGPR to regularly irrigated soils produced the highest protein content (7.18 ± 0.69 and 7.30 ± 0.74 cm; Figure 4), moisture content (8.88 ± 0.81 and 9.20 ± 0.62 cm; Figure 4), seed yield (161.69 ± 13.37 and 169.70 ± 12.35 g; Figure 4), and saponin content (51.18 ± 4.23 and 53.72 ± 3.91 t ha^−1^; Figure 4) in the 2019–2020 and 2020–2021 seasons, respectively. Similarly, the combined application of phosphogypsum and PGPR to deficiently irrigated soils produced the second highest protein content (6.36 ± 0.55 and 6.57 ± 0.49 cm; Figure 4), moisture content (7.40 ± 0.32 and 7.85 ± 0.37 cm; Figure 4), and saponin content (51.18 ± 4.23 and 53.72 ± 3.91 t ha^−1^; Figure 4) in the 2019–2020 and 2020–2021 seasons, respectively. Control quinoa plants had the lowest seed protein content, moisture content, and saponin content during both seasons.

## 3. Discussion

### 3.1. Effect of Soil Treatments on Soil Exchangeable Na Percentage, Soil Enzymes’ Activity (Urease and Dehydrogenase), in Addition to Na^+^ and K^+^ Ions in Leaves under Abiotic Stress

Water stress and soil salinity could expedite the increment of osmotic stress and was recognized as the mean abiotic factors to influence productivity by plummeting water absorbency to constrain morphological and physiological processes, leading to cell dehydration and reduction in the final germination percentage due to ions toxicity, which result in the decrease in seed germination [34]. However, combined application of phosphogypsum and PGPR minimized the harmful impact of both salinity and water stress, which promoted the germination percentage as well as plant growth and development [35].

Water stress and soil salinity together could increase exchangeable sodium percentage, which constrains the uptake of Ca^2+^ and K^+^ that cause nutrient disorder. In addition to this area was an excess of sodium and not have enough water for full irrigation. Therefore, individual application of phosphogypsum, PGPR, or both, resulted in excrete of polysaccharides from soil microbes, which is the first step in soil reclamation by decline of Na^+^ that increases the phytoavailability of most nutrients such as Ca^2+^ and K^+^, and improves soil structure and soil physical properties. The dual impact of phosphogypsum and PGPR on soil biota and their activities is due to an increase in its biostimulation impact [36], which improves the activity of soil microbes in the rhizosphere, which reflects positively on seed germination and plant development, and could provide a comfortable condition for microbial activity under abiotic stress. Urease is produced by soil microorganisms that facilitates the breakdown of the urea molecule, transforming it back into ammonia, carbon dioxide, and water, causing high losses of N to the atmosphere. However, the dehydrogenase activity in soil reflects the total oxidative activity of the microbiota, and as it is intracellular with low activity when in the free state in the soil, it can act as a good indicator of the microbial activity present in the soil [37]. The higher activities of urease and dehydrogenase, due to phosphogypsum and PGPR, increase the phytoavailability of most nutrients, physicochemical properties, and soil quality under abiotic stress [38]. In arid and semi-arid regions, there is not enough water for full irrigation, which would certainly affect these pigments, and full irrigation may not be the solution. Our results proved that the singular application of phosphogypsum or PGPR decreased Na^+^ uptake in leaves, which resulted in increased K^+^ uptake and K^+^/Na^+^ ratio. The beneficial effect of phosphogypsum or PGPR augmented the K^+^/Na^+^ ratio due to the excretion of IAA and bacterial exopolysaccharide, which can bind to Na^+^ and reduce its uptake and accumulation in plant tissues, which reflects positively on plant health under abiotic stress [39].

### 3.2. Effect of Soil Treatments on Photosynthetic Pigment Contents and Indicators of Plant Responses to Stresses under Abiotic Stress

Chlorophyll a, b, and carotenoids in leaves usually declined linearly with saline soil under water stress down to the valley, which negatively related with plant growth and development. It was found that the application of phosphogypsum or PGPR obviously improved chlorophyll a, b, and carotenoids in leaves under saline soils and water stress together, which help in cell elongation and division [40]. The combined application of phosphogypsum and PGPR gave higher chlorophyll a, b, and carotenoids contents in leaves. The increase in chlorophyll and carotenoids content in leaves was due to stifled Na and increased Ca and Mg in soil solution as well as decreased Na content and increased K content in the leaves, which helped nutrient uptake and caused water uptake to be easier [41].

Relative water content, proline content, and electrolyte leakage were recognized as indicators for estimating plant salt tolerance and water stress. Considering that the amount of electrolyte leakage is related to membrane permeability, this trait indicates damage to membranes; in other words, tolerance to stress in general. Therefore, it was found that these three factors were negatively affected by soil salinity and water stress due to the damage of the chlorophyll mechanism, the destruction of the photosynthetic system, the lack of water absorption, and the disturbance of soil nutrients and their transport to different plant organs [42]. However, application of phosphogypsum or PGPR improved the meristematic activity that causes an increase in cell division and enlargement. The improvement of relative water content, proline content, and electrolyte leakage can be ascribed to the beneficial role of phosphogypsum or PGPR that augment nutrient availability and uptake as well as water uptake [43]. It was noted that the coupled addition of phosphogypsum and PGPR for quinoa under abiotic stressors improved soil water holding capacity and plant bioavailable water, so water can absorb gradually, leading to better augmentation of RWC in the plant leaves compared to the single application [44]. Therefore, the combined application of phosphogypsum and PGPR seems to be applicable in plummeting Na uptake and improving plant water balance, resulting in the improvement of relative water content, proline content, and electrolyte leakage in quinoa plants under abiotic stress [45].

### 3.3. Effect of Soil Treatments on Antioxidant Enzymes’ Activity and Oxidative Stress under Abiotic Stress

In the present study, under water stress and soil salinity together, the antioxidant enzymatic activities, such as SOD, CAT, and POX, were increased in quinoa plants. However, this increase was not adequate to detoxify the injurious effects of ROS. Contrariwise, the singular addition of phosphogypsum led to an obvious increase in SOD, CAT, and POX activities [46]. However, it was observed that SOD, CAT, and POX activities are more boosted under the combined application of phosphogypsum and PGPR, which may be due to lessened Na uptake [47]. SOD, CAT, and POX activities could convert H_2_O_2_ into non-toxic compounds such as H_2_O and O_2_, therefore protecting the plants from their damaging effects on cell membranes and macromolecules [48].

Under the same abiotic stress, high H_2_O_2_ levels resulting in membrane degradation and lipid peroxidation were noted, which increase oxidative damage [49]. From our data, it was stated that the singular addition of phosphogypsum or PGPR under water stress and soil salinity resulted in a distinguished decrease in H_2_O_2_ contents, and a decline in lipid peroxidation (MDA) compared to unamended treatment (control) [50]. However, the coupled application of phosphogypsum and PGPR were more influential in amending oxidative stress than the singular addition, which led to transforming ROS into less or non-toxic compounds [51]. This is a clear indication of the ameliorative role of the combined application of phosphogypsum and PGPR in declining oxidative stress in quinoa plants exposed to water stress and soil salinity together.

### 3.4. Effect of Soil Amendments on Quinoa Yield Related Traits, Productivity, and Seed Quality under Abiotic Stress

Grain yield is the most important variable, considering its use in the market. The decrease in plant height (cm), 1000-grain weight (gm), grain yield, foliage yields, biological yield, and harvest index as well as qualities such as grain protein content and saponin content have been adversely influenced under water stress and soil salinity due to the decline in the incorporation and transfer of the nutrition materials through grain maturity and their filling periods. In addition, soil salinity and water stress can prompt severe damage to the ovary, and consequently may result in a decline in productivity [52]. However, the combined application of phosphogypsum and PGPR were more efficient to improve quinoa yield components and quality such as plant height (cm), 1000-grain weight (gm), grain yield, foliage yields, biological yield, and harvest index as well as qualities such as grain protein content and saponin content, as a consequence of alleviating the injurious effect of water stress and soil salinity compared to untreated plants (control treatment), which can enhance seed sterility [53]. The high efficiency of phosphogypsum could be due to its ability to lessen osmotic damage, decline Na^+^ uptake, augment K^+^ uptake, thereby keeping healthy flag leaf, increase photosynthesis with high net assimilation rate, translocate from sources to sink alongside starch accumulation in the chloroplast, decline oxidative damage, delay senescence, improve water uptake, and augment the antioxidative capacity [54]. However, the high efficiency of PGPR could be due to its ability to give high seedling vigor with a free radical defense system [55]. In this concern, soil applications are found to augment the growth and yield of higher plants, particularly under abiotic stress [56,57]. Soil microorganisms shape global element cycles in life and death. Living soil microorganisms are a major engine of terrestrial biogeochemistry, driving the turnover of soil organic matter—Earth’s largest terrestrial carbon pool and the primary source of plant nutrients. Therefore, it is now possible to leverage a trait-based understanding of microbial life and death within improved biogeochemical models and to better predict ecosystem functioning under new climate regimes [58]. All of the aforementioned advantages were more developed by combined phosphogypsum and PGPR, which presented high seed fertility with low sterility combined with heavy seed, which resulted in high yield and quality under salt and water stressors.

## 4. Materials and Methods

### 4.1. Phosphogypsum (PG) Characterization

Phosphogypsum was obtained from a fertilizer industry factory in El-Sharkia Governorate, Egypt. Some samples were taken in the lab to be ground and passed through a 2-mm mesh and then the samples were digested using nitric acid in a microwave to measure the physical and chemical characteristics of PG. Phosphogypsum consists of gypsum as the key compound, which has higher concentrations of Ca^2+^ (27.43 meq L^−1^), P (26.43 g kg^−1^), and S (14.33%) as major elements. Moreover, the value of Mg^2+^ was (3.79 meq L^−1^), O.M was (1.01%), and CEC was (61.36 cmol kg^−1^). PG had a pH of 3.25, which was measured by pH–meter and EC 3.45 dS m^−1^ (1:5 soil/water ratio). Total Cd concentration was not detected in PG samples. In addition, the concentrations of P, K, and Al accounted for 2.45%, 0.08%, and 0.11%, respectively, which were determined by coupled plasma-optical emission spectrometry (ICP-OES) (PerkinElmer Optima 4300 DV). The application rate of PG was 9 ton ha^−1^.

#### 4.1.1. Microorganisms and Culture Conditions

Bacterial inoculants of plant growth-promoting rhizobacteria (PGPR), namely Azospirillum lipoferum SP2, Bacillus coagulans NCAIM B.01123, Bacillus circulance NCAIM B.02324, and Bacillus subtilis MF497446 were attained from Bacteriology Laboratory, Sakha Agricultural Research Station, Kafr El-Sheikh, Egypt. The standard culture circumstances were set with semi-solid malate medium for A. lipoferum [59], and with nutrient broth medium for the Bacillus strains [60]. Total counts of microbes were (56 ± 2.23 CFU × 105 g^−1^ dry soil, Bacillus); (37 ± 1.67 CFU × 105 g^−1^ dry soil, Azospirillum). Total counts were performed by pasteurization in a water bath at 70 °C for 10 min to kill the vegetative cells. The inoculation treatments were prepared as peat-based inoculums, with 30 mL of 109 CFU mL^−1^ from each culture per 60 g of sterilized carrier, which were mixed carefully with the quinoa seeds, and seed planting was performed directly after seed inoculation.

#### 4.1.2. Study Site Description and Plant Material

We performed the experiments at the Elamaar village in the region of Sidi Salem (31°07′ N latitude, 30°57′ E longitude), Kafr El-sheik Governorate, Egypt during the 2019–2020 and 2020–2021 cropping seasons, to investigate the application of phosphogypsum as soil amendment coupled with PGPR inoculation on soil, growth, physiological, and quality properties as well as yield traits and productivity of quinoa (*Chenopodium quinoa*) under water stress in salt-affected soil. Quinoa seeds (Chipaya cultivar; 12 kg ha^−1^ sowing rate) were attained from Plant Breeding Unit, Desert Research Center. Quinoa seeds were planted on 15th and 18th of November for both years of study, respectively. Soil samples were collected at depth (0–30 cm) before sowing to measure the physical and chemical characteristics in both seasons. The experimental soil was analyzed and its physical and chemical properties were as follows; soil texture was clay loam and the chemical properties in both seasons were pH (8.35 and 8.58), EC (11.14 and 12.09 dS m^−1^), O.M. (1.51 and 1.48%), field capacity (29.4 and 31.1%), Na^+^ (17.78 and 18.65 meq L^−1^), K^+^ (10.73 and 11.86 meq L^−1^), Mg^2+^ (14.76 and 16.23 meq L^−1^), Ca^2+^ (6.54 and 18.29 meq L^−1^), HCO_3_^−^ (17.22 and 19.67 meq L^−1^), and SO_4_^2−^ (19.03 and 21.15 meq L^−1^), respectively. Meteorological data of the experimental site are also presented in Table 5.

#### 4.1.3. Experimental Design and Treatments

The field experiment design was arranged in a split-plot at three replicates comprising of eight treatment combinations, i.e., two water irrigation regimes as follows: regular irrigation (four irrigations) and deficit irrigation (two irrigations) in the main plots and four soil treatments in subplots. There was a total of four treatments, comprised from (1) non-treated control, without the application of phosphogypsum or PGPR; (2) soil application of phosphogypsum; (3) inoculation with PGPR; and (4) combined application of phosphogypsum and PGPR. The subplot net area was 10.50 m^2^ (7 rows of 50 cm width and 3 m length) with plant spacing 20 cm (maximum 210 plants/each subplot). The research experiments were conducted in accordance with the most important technical recommendations contained in the Bulletin of the Ministry of Agriculture and Land Reclamation. Soil of experimental plot was prepared in the same manner as the nursery area. Prior to quinoa planting, ploughing was performed at 30 cm depth and hoeing at 15 cm. During soil preparation, the experimental soil was fertilized with phosphorus fertilizer in the form of calcium superphosphate (15.5% P_2_O_5_) at the rate of 107 kg P_2_O_5_ ha^−1^ during land preparation and potassium (K) at the rate of 120 kg ha^−1^ in the form of potassium sulfate (48% K_2_O) during seedbed preparation and before ridging, and nitrogen fertilizer was applied at a rate of 286 kg ha^−1^as ammonium nitrate (33.5%) was side dressed at two equal doses, then nitrogen fertilizer was added into two portions, half being applied before the first irrigation, while the remaining portion was applied before the second irrigation.

#### 4.1.4. Soil Measurements

##### Exchangeable Sodium Percentage (ESP) in Soil

A total of ten soil samples were selected at random from different plots of the experimental site at harvest by an auger and oven-dried to determine soil Na^+^, Ca^2+^, and Mg^2+^, which are soluble cations in soil solution (meq L^–1^) in paste extract by Atomic Absorption Spectrophotometer (AAS, PERKIN ELMER 3300) to assess soil sodium adsorption ratio (SAR). Then ESP was computed by Arshad et al. [61]:

ESP = 1.95 + 1.03 × SAR (R^2^ = 0.92)

##### Soil Enzymes’ Activity

Soil samples were collected at 60 days after seed sowing, to determine the activity of soil enzymes: dehydrogenases (Deh) as described by Öhlinger [62] by adding the soil samples on INT solution, maintaining them for 2 h at 40 °C. The iodonitro-tetrazolium formazan (INTF) was obtained with dimethyl-formamide and ethanol, and afterwards computed photometrically at 464 nm., with urease (Ure) as described by Alef and Nannipieri [63] by the quantitative estimation of ammonia by the spectrophotometric assessment at 660 nm.

### 4.2. Physiological Attributes

#### 4.2.1. Measurements of Na^+^ and K^+^ Ions in Leaves

At 75 days after seed sowing, milled five leaf samples (>1.0 g) were weighed and converted to ash with an electric oven. Then, 100 μL of HNO_3_ was applied for cation extraction. The leaf Na^+^ and K^+^ contents were assessed as mg g^−1^ dry weight by an ion chromatograph based on the method of Jackson [64].

#### 4.2.2. Photosynthetic Pigment Contents

At 75 days after seed sowing, milled five leaf samples (>1.0 g) were weighed and used to determine photosynthetic pigment contents (chlorophyll a, b and carotenoids). Pigment contents in green leaves were estimated in 80% acetone for 48 h at 4 °C. The absorbance of the filtrates was assessed at different wavelengths (663, 645, and 480 nm) by a UV-1900 BMS (Waltham, MA, USA) spectrophotometer. Pigment contents were computed based on method of Anaraki [65]. Equations were used for calculation as follow: Chl a (mg g^−1^ FW) = 12.7 (A663) − 2.69 (A645); Chl b (mg g^−1^ FW) = 25.8 (A645) − 4.68 (A663); Carotenoids (mg g^−1^ FW) = (1000 (A470) − 2.27 (Chl a) − 81.4 (Chl b))/227.

#### 4.2.3. Indicators of Plant Responses to Stresses

##### Leaf Water Relations

At 75 days after seed sowing, five green leaves were selected from the mid-section and weighed (fresh weight, FW). The fresh weight attained from each sample was >0.5 g, as recommended by Weatherly [66]. So as to attain the turgid weight (TW), five green leaves were saturated in distilled water inside a closed petri dish. Then, the saturated leaves were weighed (TW) after cleaning the water carefully from the leaf surface, then dried for 24 h at 80 °C for assessment of dry weight (DW).

$$\mathrm{Relative}\;\mathrm{water}\;\mathrm{content}\;\left(\mathrm{RWC};\;\%\right)=\frac{\left(\mathrm{FW}\;-\mathrm{DW}\right)}{\left(\mathrm{TW}\;-\mathrm{DW}\right)}\times 100$$

##### Proline Content

Proline content was assessed by the method of Bates et al. [67]. At 75 days after seed sowing, green leaves were selected from the mid-section (50 mg fresh weight), which were each extracted with 5 mL of 3% sulfosalicylic acid. A total of 1 mL extract was combined with 2 mL of a dual of glacial acetic acid and ninhydrin reagent. Then, 1 h of incubation at 90 °C for 30 min was performed. The tubes were cooled and 5 mL toluene was applied. The absorbance of the upper phase was spectrophotometrically assessed at 520 nm. Free proline content was assessed from a standard curve prepared by analytical grade proline and computed as mg g^−1^ FW.

##### Electrolyte Leakage

At 75 days after seed sowing, green leaves were selected from the mid-section and cut into segments (ca. 1 cm) and weighed. The segments were saved into 20 mL of distilled water in the test tube. The leaf fragments were washed gradually then put into a shaker (100 rpm) at room temperature and the rate of leakage was read at 15 min intervals for 60 min via a LF 92 conductivity meter. The leakage rate was measured as the slope of the line to the leaf dry weight by the method of Bajji et al. [68]. The electrolyte leakage (EL %) was calculated using equation: EL= ((EC_15_ − EC_0_))/((leaf fresh weight)) × 100, where EC_0_ represents conductivity at the initial time and EC_15_ represents conductivity after 15 min.

### 4.3. Antioxidant Enzyme Activity

At 75 days after seed sowing, green leaves were selected from the mid-section (250 mg each) and were cut into segments and homogenized in 5 mL of cold phosphate buffer (50 mM phosphate buffer pH 7.0, containing 1 mM EDTA, 1 mM phenylmethylsulfonyl fluoride, and 1% polyvinylpolypirrolidone) to utilize as an enzyme extract. Then, the mixed sample was centrifuged at 15,000× *g*, 4 °C for 30 min.

#### 4.3.1. Superoxide Dismutase Activity

(SOD: 1.15.1.1) was assessed by 50% NBT reduction assay at 560 nm, as found by Beauchamp and Fridovich [69].

#### 4.3.2. Catalase Activity

(CAT: 1.11.1.6) was assessed respecting the reaction between 50 µL enzyme extract and 12.5 mm H_2_O_2_ in company with 50 mM K-phosphate buffer (pH 7.0). The reaction started by applying H_2_O_2_ and the absorbance was examined at 240 nm for 60 s [70].

#### 4.3.3. Peroxidase Activity

(POX: 1.11.1.7) was assessed by o-phenylenediamine as a chromogenic indicator in company with H_2_O_2_ and enzyme extract at 417 nm, as found by Vetter et al. [71]. The activity of all enzymes computed as units mg^−1^ protein.

### 4.4. Oxidative Stress Markers

#### 4.4.1. Hydrogen Peroxide (H_2_O_2_)

At 75 days after seed sowing, green leaves selected from the mid-section (250 mg each) were cut into segments, and homogenization of samples (0.5 g) by liquid N_2_ and tri chloroacetic acid (TCA: 0.1%) was performed, then the samples were centrifuged (3000 rpm) for 20 min to determine H_2_O_2_, as found by Velikova et al. [72]. The reaction was prepared by applying 10 mM K-phosphate buffer (pH 7.0, 1.0 mL), potassium iodide (2 M, 1 mL), and plant extract (1 mL). The absorbance was examined at 390 nm by the model UV-160 A spectrophotometer (Shimadzu, Japan). H_2_O_2_ was computed as µmol g^−1^ FW by a standard curve.

#### 4.4.2. Lipid Peroxidation (MDA)

At 75 days after seed sowing, green leaves selected from the mid-section (250 mg each) were cut into segments and homogenization of sample (0.5 g) by liquid N2 and hydro-acetone buffer (4:1 *v*/*v*) was performed. A total of 0.65% thiobarbituric acid (TBA) and 0.01% Butyl hydroxyl toluene (BHT) were used to determine thiobarbituric acid reactive substances (TBARS), following the method found by Du and Bramlage [73], then samples were incubated at 95 °C and centrifuged (10,000× *g* ) for 15 min. The absorbance was examined at 532 and 600 nm by a spectrophotometer. MDA was computed as µmol g^−1^ FW by a standard curve.

### 4.5. Plant Growth and Yield Parameters

After maturity, canopy biomass was collected and estimated after oven drying at 70 °C until constant weight. Plant height (cm), 1000-grain weight (g), grain yield, foliage yields, and harvest index were measured from the remaining plants in each replicate. The biological yield (both grain and straw yield ton ha^-1^) was measured from a 6-m^2^ area in each experimental unit except the outer border, and the standard grain moisture content of 14% was added to yield computation. Harvest index (HI; %) was computed as the ratio between grain and biological yields and expressed as %. HI (%) was calculated using Equation:

$$\mathrm{Harvest}\;\mathrm{index},\;\%=\frac{\mathrm{Grain}\;\mathrm{yield}\;\left({\mathrm{kg}\;\mathrm{ha}}^{-1}\right)}{\mathrm{Biological}\;\mathrm{yield}\;\left({\mathrm{kg}\;\mathrm{ha}}^{-1}\right)}\times 100$$

### 4.6. Phytochemical Screening of the Extracts

During the harvesting, five plants from each treatment were sampled from each replicate, split along the stem, and oven dried for 30 min at 105 °C, and then kept for 48 h at 65 °C until constant weight. After that, the grain samples were combined, ground to a fine powder, and stored for quality analysis using a laboratory mill (model IKA A10-IKAWERKE; GmbH &CO. KG, Staufen, Germany) according to the Association of Official Analytical Chemists (AOAC) methods. The extracts obtained were subjected to preliminary phytochemical screening as follows. Grains protein (%) was estimated by multiplying the total nitrogen by 6.25 using Kjeldahl method according to [74].

Saponin content was estimated using reverse phase based on the grains for extraction (10 g), which were thoroughly ground and then extracted with water at 60 °C for 3 h. The ratio of water to grains was 15 to 1 (by weight). The extract was centrifuged and the supernatant filtered. The Saponin content was calculated as following formula:

Total saponins (%) = weight of residue × 100/weight of sample.

### 4.7. Statistical Analysis

Normality and homoscedasticity of dependent variables were checked. Data analysis was performed using the SPSS 13.0 software package (SPSS Inc., Chicago, IL, USA). The analysis of variance using two-way ANOVA was performed between water regimes and treatments ecotypes. One-way ANOVA was applied to assess the differences either among water regimes or treatments. Separation of means was performed by post-hoc test (Tukey’s test), and significant differences were accepted at the levels *p* < 0.05, 0.01, and 0.001. The data were presented as mean ± standard deviation. Spearman coefficient was run to investigate the non-parametric correlation between water regimes and treatments.

## 5. Conclusions

Our results investigate that coupled phosphogypsum soil amendment and seed inoculation using PGPR can be an effective, sustainable, and ecological strategy to enhance the resistance of quinoa plants grown under deficiently irrigation in salt-affected soils, particularly in arid and semi-arid zones. Coupled application of phosphogypsum soil amendment and seed inoculation using PGPR significantly improved the growth, physiology, productivity, seed quality, and salt stress tolerance of quinoa plants, along with soil characterization and nutrient absorption in 2019–2020 and 2020–2021 seasons under field conditions. Our results showed that the useful role of phosphogypsum soil amendment and seed inoculation using PGPR might be owing to the activation of the enzymatic antioxidant defense machinery to scavenge reactive oxygen species (ROS) homeostasis within stressed plants. However, itis required in the future to investigate the long-term studies on the impact(s) of phosphogypsum soil amendment and seed inoculation using PGPR on both plant and soil ecosystems.

## Figures and Tables

**Table 1:** Exchangeable sodium percentage (ESP%), urease, and dehydrogenase activity in soil after treating quinoa plants grown in the presence of different water regimes with phosphogypsum, plant growth-promoting rhizobacteria, or both.

			ESP (%)	Urease (mg TPF g^−1^ Dry Soil d^−1^)	Dehydrogenase (mg NH_4_^+^ g^−1^ Dry Soil d^−1^)
**Water regime**					
Regular Irrigation_2019–2020			14.7 ± 0.37 ^c^	139.4 ± 4.2 ^b^	90.9 ± 3.6 ^b^
Deficit irrigation_2019–2020			21.1 ± 0.32 ^a^	94.1 ± 2.8 ^d^	66.1 ± 3.3 ^d^
Regular Irrigation_2020–2021			13.6 ± 0.31 ^d^	150.3 ± 4.2 ^a^	96.5 ± 3.8 ^a^
Deficit irrigation_2020–2021			19.8 ± 0.36 ^b^	100.7 ± 2.7 ^c^	75.1 ± 3.0 ^c^
**Treatment**					
Control			21.3 ± 0.35 ^a^	82.3 ± 1.8 ^d^	41.6 ± 1.2 ^d^
PGPR			15.9 ± 0.36 ^c^	125.4 ± 3.2 ^b^	90.1 ± 1.5 ^b^
Phosphogypsum			18.6 ± 0.41 ^b^	111.3 ± 2.5 ^c^	72.9 ± 1.2 ^c^
Combined			13.5 ± 0.37 ^d^	165.6 ± 3.7 ^a^	123.9 ± 1.9 ^a^
**Interaction**					
Year	Water regime	Treatment			
2019–2020	Regular Irrig.	Control	19.1 ± 0.16 ^c, D^	93.0 ± 0.14 ^f, K^	48.8 ± 0.10 ^g, M^
		PGPR	13.0 ± 0.12 ^f, J^	145.0 ± 0.42 ^b, D^	99.6 ± 0.16 ^c, F^
		Phosphogypsum	15.9 ± 0.20 ^e, G^	127.0 ± 0.19 ^d, G^	79.8 ± 0.21 ^d, H^
		Combined	10.7 ± 0.12 ^g, L^	192.8 ± 0.27 ^a, B^	135.2 ± 0.33 ^a, B^
	Deficit Irrig.	Control	24.9 ± 0.21 ^a, A^	62.9 ± 0.21 ^h, N^	26.5 ± 0.41 ^h, O^
		PGPR	19.4 ± 0.17 ^c, D^	96.2 ± 0.25 ^e, J^	72.8 ± 0.27 ^e, I^
		Phosphogypsum	22.3 ± 0.21 ^b, C^	86.3 ± 0.44 ^g, L^	58.7 ± 0.25 ^f, K^
		Combined	17.7 ± 0.18 ^d, F^	130.7 ± 0.43 ^c, F^	106.3 ± 0.28 ^b, D^
2020–2021	Regular Irrig.	Control	17.6 ± 0.12 ^d, F^	101.5 ± 0.21 ^d, H^	52.7 ± 0.02 ^f, L^
		PGPR	12.5 ± 0.17 ^g, K^	160.0 ± 0.26 ^b, C^	104.4 ± 0.36 ^c, E^
		Phosphogypsum	14.3 ± 0.04 ^f, I^	137.9 ± 0.24 ^c, E^	84.3 ± 0.45 ^d, G^
		Combined	10.2 ± 0.18 ^h, M^	201.9 ± 0.58 ^a, A^	144.5 ± 0.39 ^a, A^
	Deficit Irrig.	Control	23.5 ± 0.08 ^a, B^	71.9 ± 0.63 ^g, M^	38.3 ± 0.46 ^g, N^
		PGPR	18.5 ± 0.04 ^c, E^	100.3 ± 0.02 ^e, I^	83.6 ± 0.32 ^d, G^
		Phosphogypsum	21.8 ± 0.17 ^b, C^	94.0 ± 0.05 ^f, K^	69.1 ± 1.00 ^e, J^
		Combined	15.5 ± 0.12 ^e, H^	136.8 ± 0.43 ^c, E^	109.5 ± 0.64 ^b, C^
**F-value (Two-way)**					
Water regime			6999.15 ***	79,968.43 ***	13,608.91 ***
Treatment			5815.84 ***	123,147.72 ***	81,585.23 ***
Interaction			32.53 ***	1891.50 ***	305.76 ***

Means in the same column within the same season followed by the different lowercase letters are significant according to the Tukey’s test (*p* ≤ 0.05) and different uppercase letters are significant regardless of the growing season according to the Tukey’s test (*p* ≤ 0.05). Data are Means ± SD and n = 3. *** denotes significance at *p* ≤ 0.001.

**Table 2:** Uptake of Na^+^, K^+^, and K^+^/Na^+^ ratio in leaves of quinoa plants grown in the presence of different water regimes and treated with phosphogypsum, plant growth-promoting rhizobacteria, or both.

			Na^+^ (mg g^−1^ DW)	K^+^ (mg g^−1^ DW)	K^+^/Na^+^
**Water regime**					
*Regular Irrigation_2019–2020*			3.15 ± 0.04 ^b^	3.81 ± 0.05 ^a^	1.24 ± 0.03 ^a^
*Deficit irrigation_2019–2020*			3.90 ± 0.04 ^a^	3.00 ± 0.03 ^c^	0.78 ± 0.02 ^b^
*Regular Irrigation_2020–2021*			3.23 ± 0.04 ^b^	3.87 ± 0.05 ^a^	1.23 ± 0.03 ^a^
*Deficit irrigation_2020–2021*			3.92 ± 0.04 ^a^	3.11 ± 0.03 ^b^	0.81 ± 0.02 ^b^
**Treatment**					
*Control*			4.01 ± 0.04 ^a^	2.97 ± 0.03 ^d^	0.75 ± 0.02 ^d^
*PGPR*			3.68 ± 0.04 ^b^	3.32 ± 0.05 ^c^	0.92 ± 0.02 ^c^
*Phosphogypsum*			3.43 ± 0.04 ^c^	3.53 ± 0.05 ^b^	1.06 ± 0.03 ^b^
*Combined*			3.09 ± 0.04 ^d^	3.97 ± 0.06 ^a^	1.32 ± 0.03 ^a^
**Interaction**					
*Year*	*Water regime*	*Treatment*			
2019–2020	Regular Irrig.	Control	3.59 ± 0.10 ^c, DE^	3.18 ± 0.07 ^e, FG^	0.89 ± 0.04 ^e, E^
		PGPR	3.27 ± 0.04 ^d, FG^	3.69 ± 0.10 ^c, CD^	1.13 ± 0.04 ^c, C^
		Phosphogypsum	3.00 ± 0.06 ^e, HI^	3.97 ± 0.10 ^b, B^	1.33 ± 0.02 ^b, B^
		Combined	2.73 ± 0.06 ^f, J^	4.39 ± 0.02 ^a, A^	1.61 ± 0.03 ^a, A^
	Deficit Irrig.	Control	4.36 ± 0.07 ^a, A^	2.68 ± 0.03 ^f, J^	0.62 ± 0.01 ^h, I^
		PGPR	4.02 ± 0.11 ^b, BC^	2.83 ± 0.01 ^f, IJ^	0.71 ± 0.02 ^g, GHI^
		Phosphogypsum	3.81 ± 0.06 ^b, CD^	3.05 ± 0.04 ^e, GH^	0.80 ± 0.02 ^f, EFG^
		Combined	3.40 ± 0.08 ^cd, ED^	3.44 ± 0.04 ^d, E^	1.01 ± 0.01 ^d, D^
2020–2021	Regular Irrig.	Control	3.69 ± 0.09 ^c, D^	3.24 ± 0.03 ^e, F^	0.88 ± 0.01 ^d, E^
		PGPR	3.36 ± 0.06 ^de, EFG^	3.77 ± 0.12 ^c, C^	1.12 ± 0.05 ^c, C^
		Phosphogypsum	3.12 ± 0.07 ^e, GH^	3.99 ± 0.02 ^b, B^	1.28 ± 0.03 ^b, B^
		Combined	2.77 ± 0.11 ^f, IJ^	4.49 ± 0.09 ^a, A^	1.62 ± 0.09 ^a, A^
	Deficit Irrig.	Control	4.39 ± 0.08 ^a, A^	2.77 ± 0.04 ^g, J^	0.63 ± 0.02 ^f, HI^
		PGPR	4.08 ± 0.09 ^b, B^	2.99 ± 0.02 ^f, HI^	0.73 ± 0.01 ^ef, FGH^
		Phosphogypsum	3.79 ± 0.09 ^c, CD^	3.13 ± 0.02 ^ef, FGH^	0.83 ± 0.02 ^de, EF^
		Combined	3.43 ± 0.08 ^d, EF^	3.55 ± 0.04 ^d, DE^	1.04 ± 0.02 ^c, CD^
**F-value (Two-way)**					
Water regime			329.31 ***	710.52 ***	562.93 ***
Treatment			287.38 ***	596.16 ***	504.28 ***
Interaction			0.42 ns	12.40 ***	14.80 ***

Means in the same column within the same season followed by the different lowercase letters are significant according to the Tukey’s test (*p* ≤ 0.05) and different uppercase letters are significant regardless of the growing season according to the Tukey’s test (*p* ≤ 0.05). Data are Means ± SD and n = 3. *** denotes significance at *p* ≤ 0.001, ns denotes non-significant.

**Table 3:** Contents of photosynthetic pigments in quinoa leaves grown in the presence of different water regimes and treated with phosphogypsum, plant growth-promoting rhizobacteria, or both.

			chl_a (mg g^−1^ FW)	chl_b (mg g^−1^ FW)	Carotenoids (mg g^−1^ FW)
**Water regime**					
*Regular Irrigation_2019–2020*			0.480 ± 0.06 ^b^	0.722 ± 0.05 ^b^	0.913 ± 0.01 ^b^
*Deficit irrigation_2019–2020*			0.370 ± 0.05 ^d^	0.620 ± 0.05 ^d^	0.638 ± 0.01 ^d^
*Regular Irrigation_2020–2021*			0.494 ± 0.07 ^a^	0.744 ± 0.07 ^a^	0.966 ± 0.01 ^a^
*Deficit irrigation_2020–2021*			0.384 ± 0.06 ^c^	0.633 ± 0.06 ^c^	0.684 ± 0.02 ^c^
**Treatment**					
*Control*			0.365 ± 0.05 ^d^	0.615 ± 0.05 ^d^	0.634 ± 0.02 ^d^
*PGPR*			0.410 ± 0.07 ^c^	0.659 ± 0.07 ^c^	0.788 ± 0.02 ^c^
*Phosphogypsum*			0.449 ± 0.07 ^b^	0.699 ± 0.07 ^b^	0.843 ± 0.02 ^b^
*Combined*			0.504 ± 0.07 ^a^	0.746 ± 0.07 ^a^	0.936 ± 0.01 ^a^
**Interaction**					
*Year*	*Water regime*	*Treatment*			
2019–2020	Regular Irrig.	Control	0.402 ± 0.004 ^e, H^	0.653 ± 0.004 ^e, G^	0.763 ± 0.012 ^c, GH^
		PGPR	0.469 ± 0.006 ^c, E^	0.719 ± 0.006 ^c, DE^	0.913 ± 0.007 ^b, DE^
		Phosphogypsum	0.495 ± 0.007 ^b, D^	0.745 ± 0.005 ^b, C^	0.953 ± 0.051 ^b, CD^
		Combined	0.554 ± 0.007 ^a, B^	0.770 ± 0.009 ^a, B^	1.023 ± 0.015 ^a, AB^
	Deficit Irrig.	Control	0.317 ± 0.001 ^h, K^	0.567 ± 0.001 ^h, J^	0.477 ± 0.005 ^e, J^
		PGPR	0.347 ± 0.004 ^g, J^	0.597 ± 0.004 ^g, I^	0.623 ± 0.012 ^d, I^
		Phosphogypsum	0.382 ± 0.001 ^f, I^	0.632 ± 0.001 ^f, H^	0.670 ± 0.026 ^d, I^
		Combined	0.434 ± 0.004 ^d, G^	0.684 ± 0.004 ^d, F^	0.780 ± 0.017 ^c, GH^
2020–2021	Regular Irrig.	Control	0.413 ± 0.001 ^e, H^	0.663 ± 0.001 ^e, G^	0.820 ± 0.017 ^d, FG^
		PGPR	0.473 ± 0.002 ^c, E^	0.723 ± 0.002 ^c, D^	0.960 ± 0.018 ^c, CD^
		Phosphogypsum	0.515 ± 0.004 ^b, C^	0.765 ± 0.004 ^b, B^	1.003 ± 0.021 ^b, BC^
		Combined	0.577 ± 0.009 ^a, A^	0.827 ± 0.009 ^a, A^	1.080 ± 0.010 ^a, A^
	Deficit Irrig.	Control	0.327 ± 0.006 ^g, K^	0.577 ± 0.006 ^f, J^	0.477 ± 0.012 ^g, J^
		PGPR	0.353 ± 0.004 ^f, J^	0.596 ± 0.013 ^f, I^	0.657 ± 0.015 ^f, I^
		Phosphogypsum	0.405 ± 0.005 ^e, H^	0.655 ± 0.005 ^e, G^	0.743 ± 0.015 ^e, H^
		Combined	0.453 ± 0.007 ^d, F^	0.703 ± 0.007 ^d, E^	0.860 ± 0.010 ^d, EF^
**F-value (Two-way)**					
Water regime			2012.95 ***	1300.20 ***	850.41 ***
Treatment			1708.64 ***	1040.72 ***	508.04 ***
Interaction			14.62 ***	16.48 ***	5.33 ***

Means in the same column within the same season followed by the different lowercase letters are significant according to the Tukey’s test (*p* ≤ 0.05) and different uppercase letters are significant regardless of the growing season according to the Tukey’s test (*p* ≤ 0.05). Data are Means ± SD and n = 3. *** denotes significance at *p* ≤ 0.001.

**Table 4:** Yield and yield components of quinoa plants grown in the presence of different water regimes and treated with phosphogypsum, plant growth-promoting rhizobacteria, or both.

			Plant Height, cm	1000-Seed Weight (gm)	Seed Yield (t ha^−1^)	Foliage Yield (t ha^−1^)	Biological Yield ^‡^ (t ha^−1^)	Harvest Index (%)
**Water regime**								
*Regular Irrigation_2019–2020*			142.4 ± 3.94 ^b^	3.49 ± 0.11 ^a^	2.93 ± 0.06 ^b^	4.43 ± 0.06 ^b^	7.36 ± 0.11 ^b^	39.6 ± 0.16 ^b^
*Deficit irrigation_2019–2020*			83.3 ± 2.84 ^d^	1.91 ± 0.05 ^b^	1.98 ± 0.05 ^d^	3.48 ± 0.05 ^d^	5.47 ± 0.09 ^d^	36.0 ± 0.24 ^d^
*Regular Irrigation_2020–2021*			151.4 ± 4.65 ^a^	3.44 ± 0.14 ^a^	3.10 ± 0.06 ^a^	4.60 ± 0.06 ^a^	7.71 ± 0.12 ^a^	40.1 ± 0.16 ^a^
*Deficit irrigation_2020–2021*			89.0 ± 2.69 ^c^	1.84 ± 0.04 ^c^	2.17 ± 0.05 ^c^	3.67 ± 0.05 ^c^	5.85 ± 0.09 ^c^	36.9 ± 0.20 ^c^
**Treatment**								
*Control*			80.6 ± 2.72 ^d^	1.74 ± 0.05 ^d^	1.94 ± 0.04 ^d^	3.44 ± 0.04 ^d^	5.39 ± 0.09 ^d^	35.8 ± 0.23 ^d^
*PGPR*			122.9 ± 4.28 ^b^	2.94 ± 0.10 ^b^	2.38 ± 0.06 ^c^	3.88 ± 0.06 ^c^	6.25 ± 0.12 ^c^	37.7 ± 0.24 ^c^
*Phosphogypsum*			100.5 ± 2.64 ^c^	2.25 ± 0.07 ^c^	2.67 ± 0.06 ^b^	4.17 ± 0.06 ^b^	6.83 ± 0.12 ^b^	38.8 ± 0.19 ^b^
*Combined*			162.2 ± 4.55 ^a^	3.75 ± 0.15 ^a^	3.21 ± 0.06 ^a^	4.71 ± 0.06 ^a^	7.91 ± 0.12 ^a^	40.3 ± 0.15 ^a^
**Interaction**								
*Year*	*Water regime*	*Treatment*						
2019–2020	Regular Irrig.	Control	101.1 ± 0.37 ^e, J^	2.34 ± 0.051 ^de, GH^	2.25 ± 0.026 ^d, G^	3.75 ± 0.026 ^d, G^	6.00 ± 0.053 ^e, F^	37.5 ± 0.11 ^d, F^
		PGPR	151.7 ± 0.26 ^b, D^	3.70 ± 0.064 ^b, D^	2.79 ± 0.040 ^c, EF^	4.29 ± 0.040 ^c, EF^	7.08 ± 0.080 ^c, E^	39.4 ± 0.12 ^c, DE^
		Phosphogypsum	124.2 ± 0.11 ^c, F^	3.01 ± 0.103 ^c, E^	3.09 ± 0.068 ^b, CD^	4.59 ± 0.068 ^b, CD^	7.67 ± 0.050 ^b, CD^	40.2 ± 0.17 ^b, BC^
		Combined	192.7 ± 0.12 ^a, B^	4.91 ± 0.104 ^a, B^	3.58 ± 0.084 ^a, B^	5.08 ± 0.090 ^a, B^	8.67 ± 0.050 ^a, B^	41.3 ± 0.33 ^a, A^
	Deficit Irrig.	Control	52.7 ± 0.30 ^h, O^	1.28 ± 0.041 ^g, L^	1.49 ± 0.078 ^g, J^	2.99 ± 0.078 ^g, J^	4.49 ± 0.155 ^h, I^	33.3 ± 0.27 ^g, I^
		PGPR	86.0 ± 0.10 ^f, K^	2.18 ± 0.042 ^e, HI^	1.75 ± 0.031 ^f, I^	3.25 ± 0.031 ^f, I^	4.99 ± 0.061 ^g, H^	35.0 ± 0.18 ^f, H^
		Phosphogypsum	74.2 ± 0.20 ^g, M^	1.69 ± 0.036 ^f, K^	2.05 ± 0.061 ^e, H^	3.55 ± 0.061 ^e, H^	5.60 ± 0.122 ^f, G^	36.6 ± 0.29 ^e, G^
		Combined	120.5 ± 0.44 ^d, H^	2.50 ± 0.012 ^d, FG^	2.65 ± 0.049 ^c, F^	4.15 ± 0.049 ^c, F^	6.79 ± 0.099 ^d, E^	39.0 ± 0.16 ^c, E^
2020–2021	Regular Irrig.	Control	106.7 ± 0.05 ^e, I^	2.02 ± 0.016 ^e, IJ^	2.37 ± 0.017 ^e, G^	3.87 ± 0.017 ^e, G^	6.24 ± 0.035 ^e, F^	38.0 ± 0.07 ^e, F^
		PGPR	167.3 ± 0.43 ^b, C^	3.88 ± 0.073 ^b, C^	2.95 ± 0.061 ^c, DE^	4.45 ± 0.061 ^c, DE^	7.40 ± 0.122 ^c, D^	39.9 ± 0.17 ^c, CD^
		Phosphogypsum	122.1 ± 0.10 ^d, G^	2.63 ± 0.029 ^c, F^	3.23 ± 0.036 ^b, C^	4.73 ± 0.036 ^b, C^	7.96 ± 0.072 ^b, C^	40.6 ± 0.09 ^b, B^
		Combined	209.5 ± 0.27 ^a, A^	5.21 ± 0.027 ^a, A^	3.87 ± 0.015 ^a, A^	5.37 ± 0.015 ^a, A^	9.23 ± 0.031 ^a, A^	41.9 ± 0.03 ^a, A^
	Deficit Irrig.	Control	62.0 ± 0.10 ^h, N^	1.34 ± 0.069 ^g, L^	1.66 ± 0.049 ^g, I^	3.16 ± 0.049 ^g, I^	4.83 ± 0.099 ^g, H^	34.5 ± 0.31 ^g, H^
		PGPR	86.6 ± 0.17 ^f, K^	1.99 ± 0.064 ^e, J^	2.01 ± 0.049 ^f, H^	3.51 ± 0.049 ^f, H^	5.53 ± 0.099 ^f, G^	36.4 ± 0.24 ^f, G^
		Phosphogypsum	81.4 ± 0.38 ^g, L^	1.67 ± 0.053 ^f, K^	2.29 ± 0.045 ^e, G^	3.79 ± 0.045 ^e, G^	6.09 ± 0.090 ^e, F^	37.7 ± 0.18 ^e, F^
		Combined	126.1 ± 0.38 ^c, E^	2.35 ± 0.035 ^d, G^	2.73 ± 0.085 ^d, F^	4.23 ± 0.085 ^d, F^	6.95 ± 0.170 ^d, E^	39.2 ± 0.24 ^d, E^
**F-value (Two-way)**								
Water regime			204,669.72 ***	3043.54 ***	1247.59 ***	1221.45 ***	1604.70 ***	1146.44 ***
Treatment			200,957.53 ***	2722.49 ***	1151.69 ***	1127.57 ***	1481.35 ***	1033.50 ***
Interaction			4679.00 ***	203.77 ***	8.71 ***	8.53 ***	11.20 ***	13.27 ***

**^‡^** Biological yield is sum of seed yield and foliage yield. Means in the same column within the same season followed by the different lowercase letters are significant according to the Tukey’s test (*p* ≤ 0.05) and different uppercase letters are significant regardless of the growing season according to the Tukey’s test (*p* ≤ 0.05). Data are Means ± SD and n = 3. *** denotes significance at *p* ≤ 0.001.

**Table 5:** Meteorological data of the experimental sites during 2019–2020 and 2020–2021 growing seasons.

	Year	2019–2020				2020–2021			
		Temperature (°C)		Rainfall (mm)	Relative Humidity (%)	Temperature (°C)		Rainfall (mm)	Relative Humidity (%)
Month		Max	Min			Max	Min		
October		26.3	17.2	0.98	32.6	25.3	16.2	0.94	31.6
December		25.9	15.3	0.85	34.2	24.9	14.3	0.82	33.2
January		24.5	13.2	1.1	35.1	23.2	12.4	0.54	32.7
February		22.3	10.3	3.1	46.2	20.3	11.1	3.32	42.4
March		21.4	9.7	6.4	44.3	20.6	10.7	6.85	43.1
April		23.7	13.8	0.5	43.8	22.5	12.5	0.63	44.8

Meteorological data were obtained from Sakha Station, Kafrelsheikh Governorate, Egypt.

## Data Availability

The data that supports the findings of this study are contained within the article and available from the corresponding author upon reasonable request.
